# Predictors for an unsuccessful INtubation-SURfactant-Extubation procedure: a cohort study

**DOI:** 10.1186/1471-2431-14-155

**Published:** 2014-06-19

**Authors:** Nis Brix, Anna Sellmer, Morten Søndergaard Jensen, Linda Vad Pedersen, Tine Brink Henriksen

**Affiliations:** 1 Perinatal Epidemiology Research Unit, Aarhus University Hospital, Brendstrupgaardsvej 100, Aarhus N 8200, Denmark; 2 Department of Pediatrics, Aarhus University Hospital, Brendstrupgaardsvej 100, Aarhus N 8200, Denmark

**Keywords:** Respiratory distress syndrome, Pulmonary surfactants, Premature neonates, Mechanical ventilation, Continuous positive airway pressure

## Abstract

**Background:**

The INtubation-SURfactant-Extubation (INSURE) is a procedure that is increasingly being used to treat the respiratory distress syndrome in preterm infants. The objective of this study was to identify predictors for an unsuccessful INSURE procedure.

**Methods:**

The neonates included were less than 32 weeks’ gestation, treated with surfactant in the neonatal intensive care unit, and born 1998–2010. INSURE was defined as surfactant administration during intubation for less than 2 hours without the need for mechanical ventilation. INSURE success was defined as no re-intubation within 72 hours after INSURE, and INSURE failure was defined as re-intubation within 72 hours after INSURE. An unsuccessful INSURE procedure was either INSURE failure or mechanical ventilation for more than 24 hours immediately after surfactant administration. All predictors were defined a priori and were present before surfactant administration. Multivariate logistic regression was performed.

**Results:**

In total, 322 neonates were included: 31% (n = 100) had INSURE success, 10% (n = 33) had INSURE failure, 49% (n = 158) needed mechanical ventilation for more than 24 hours, and the remaining 10% (n = 31) needed mechanical ventilation for less than 24 hours. Predictors for INSURE failure were low gestational age and hemoglobin below 8.5 mmol/l. Predictors for mechanical ventilation for more than 24 hours were low gestational age, Apgar at 5 minutes below 7, oxygen need above 50%, CO_2_ pressure above 7 kPa (~53 mmHg), pH below 7.3, lactate above 2.5 mmol/l, need for inotropes, and surfactant administration shortly after birth, whereas preeclampsia reduced the risk.

**Conclusions:**

We identified specific predictors associated with an unsuccessful INSURE procedure. Keeping high-risk neonates with one or several predictors intubated and treated with mechanical ventilation after surfactant may prevent a re-intubation procedure.

## Background

The respiratory distress syndrome (RDS) can be treated according to the INtubation-SURfactant-Extubation (INSURE) procedure [1]. However, INSURE failure, with the need for re–intubation and mechanical ventilation (MV), is common [2-7]. Keeping neonates at high risk of INSURE failure intubated on MV after surfactant may prevent a re-intubation procedure, which may decrease the morbidity and mortality of the neonates because re-intubation is associated with airway injuries [8], blood pressure deviations, and post-extubation atelectasis [9].

No randomized controlled trials have directly evaluated the efficacy of INSURE in extremely preterm neonates (<28 weeks gestation) [1]. In some of these infants treatment with MV after surfactant might be a better option due to the high risk of INSURE failure. When a neonate with RDS needs surfactant in our department, the attending neonatologist decides, based on clinical evaluation, whether the neonate should be treated according to INSURE or with MV immediately after surfactant. Infants experiencing INSURE failure and infants needing MV for more than 24 hours immediately after surfactant can be regarded as having had an *unsuccessful INSURE procedure*.

Two studies have assessed predictors of INSURE failure [5,6]. The authors report that an increased risk of INSURE failure was found in neonates with low birth weight, high partial pressure of carbon dioxide (pCO_2_), low arterial-alveolar oxygen tension ratio (a/A-ratio), severe RDS on chest x-ray, and low partial pressure of arterial oxygen to fraction of inspired oxygen ratio (pO_2_/FiO_2_-ratio) [5,6]. We report here findings in a considerably larger study population than in the two studies mentioned above, and we are the first to suggest that predictors for MV for more than 24 hours might also predict INSURE failure.

In this paper we identify predictors associated with an unsuccessful INSURE procedure defined as either INSURE failure or MV for more than 24 hours, in contrast to the two papers that describe INSURE failure [5,6].

## Methods

The Danish National Board of Health and the Danish Data Protection Agency approved the study. We conducted a historical cohort study based on data from medical records concerning admissions to the neonatal intensive care unit (NICU) and on the Aarhus Birth Cohort (ABC), which has been described in detail elsewhere [10,11]. In brief, the ABC included all women scheduled for delivery at Aarhus University Hospital, Denmark (AUH), and contains information on delivery, clinical state of the neonate immediately after birth, and information on neonates admitted to the NICU (complete from 1998). Using the ABC, neonates with the following characteristics were identified: born 1998–2010, born at AUH, born prior to 32 completed weeks of gestation, and treated with surfactant. Neonates receiving surfactant as part of emergency intubation for resuscitation in the delivery room were excluded.

The predictors based on information from the ABC included antenatal steroids, cesarean delivery vs. vaginal delivery, preeclampsia, prolonged rupture of membranes >18 hours, maternal age at delivery, parity, multiple pregnancy, gender, Apgar score 1 and 5 minutes after birth, and umbilical cord standard base excess (SBE) and pH. Predictors obtained from the medical records included gestational age, birth weight, small for gestational age (SGA) defined as below the 10th percentile according to the formula developed by Marsal et al. [12], first temperature within 6 hours of admission to the NICU, last mean arterial blood pressure within 4 hours before intubation, hours from birth to surfactant administration, and whether inotropes were given prior to intubation. The following predictors were collected within 2 hours before intubation: FiO_2_, pO_2_ (transcutaneous or arterial), pCO_2_ (transcutaneous, capillary or arterial), and a/A-ratio estimated by the formula: arterial pO_2_ in kPa/(95 × FiO_2_ – arterial pCO_2_ in kPa). The following predictors were collected within 5 hours before intubation: pH, SBE, lactate, and hemoglobin. Predictors were present prior to surfactant administration (i.e., prior to the knowledge of the need for intubation). Furthermore, predictors were defined prior to analyses based on former studies and clinical experience [5,6].

Post-surfactant characteristics were identified but were not in strict sense present before surfactant administration in all neonates and were therefore not regarded as predictors. Post-surfactant characteristics were the clinical risk index for babies [13], infusions given within 6 hours after surfactant including inotropes, packed red blood cells, and volume expansion, antibiotics given for 7 days or more and started within 24 hours after birth, necrotizing enterocolitis [14], patent ductus arteriosus, retinopathy of prematurity grade III [15], periventricular leukomalasia [16], intraventricular hemorrhage [17], and the following respiratory variables: duration of MV, duration of nasal continuous positive airway pressure (nCPAP), duration of supplemental oxygen, neonatal mortality, and BPD. BPD was defined as needing supplemental oxygen, nCPAP, or MV at 36 post-menstrual weeks. The neonate had no BPD if transferred (without supplemental oxygen, nCPAP, or MV) or discharged before 36 post-menstrual weeks. BPD was regarded as missing if transferred before 36 post-menstrual weeks with supplemental oxygen, nCPAP, or MV. If the neonate died before 36 post-menstrual weeks, the neonate was not included in analyses of BPD. Data entry was done using EpiData 3.1 software (Odense, Denmark).

INSURE was defined as surfactant administration during intubation for less than 2 hours without the need for mechanical ventilation. INSURE success was defined as no re-intubation within 72 hours after INSURE, and INSURE failure was defined as re-intubation within 72 hours after INSURE. Neonates mechanically ventilated for ≤24 hours were classified as short MV, and neonates mechanically ventilated more than 24 hours was classified as long MV. Non-mechanically ventilated neonates that were intubated for more than 2 hours during surfactant administration were defined as short MV (n = 1). Neonates with initial MV ≤24 hours that were re-intubated within 72 hours after extubation were defined as long MV (n = 7). Neonates treated with INSURE or MV for ≤24 hours who died within 72 hours after extubation were categorized as follows: neonates who were extubated to receive palliative care (and thus died shortly after extubation) within 2 hours after the intubation-procedure with surfactant were defined as long MV (n = 1), assuming they would have required MV for more than 24 hours to survive; neonates treated with INSURE who died within 72 hours after extubation were defined as INSURE failures (n = 1), assuming they would have needed re-intubation and MV to survive; neonates treated with MV for ≤24 hours who were extubated to palliative care (n = 10) or who died within 72 hours after extubation (n =2) were defined as long MV, assuming they would have needed continued MV for more than 24 hours or re-intubation to survive. All groups were defined prior to the analyses.

In our unit, the indication for surfactant administration was an a/A-ratio <0.22 for neonates with a gestational age of 30 weeks or more and an a/A-ratio <0.36 for neonates with a gestational age below 30 weeks according to the studies by Verder et al. 1994 and 1999 [7,18]. This corresponds to an FiO_2_ ≥ 0.55 and an FiO_2_ ≥ 0.35, respectively [3]. The administered surfactant was *Poractant Alfa* (Curosurf®, Chiesi Pharmaceuticals, Parma, Italy). After delivery all neonates were stabilized on nCPAP initially on 5 cmH_2_O, with the possibility of adjustment to yield a pH above 7.30 using Benveniste’s pediatric gas-jet valve (Dameca, Copenhagen, Denmark). A loading dose of theophyllamine (theophylline and ethylenediamine in 2:1 ratio) or caffeine citrate was given immediately after admission to the NICU. All neonates received pre-intubation medication and the vast majority received the following: atropine 10–20 μg/kg, suxametonium 2 mg/kg, thiomebumal 2–5 mg/kg, and morphine 0.1 mg/kg. In case of insufficient breathing after surfactant, naloxone 0.1 mg/kg as antidote could be given. After intratracheal administration of 200 mg/kg surfactant and manual ventilation the neonate was usually rapidly extubated to nCPAP. If there was a high oxygen demand (FiO_2_ above 30%), a high transcutaneous CO_2_ (above 7.5 kPa), or a pH less than 7.20, the neonate was kept intubated and treated with MV (Babylog 800+, Dräger, Lübeck, Germany). As our cohort dates back to 1998, the optimal dosing of surfactant was not established. Therefore, some neonates suspected of needing MV after intubation were treated with an initial dose of 100 mg/kg immediately after intubation and a repeated dose of 100 mg/kg immediately after MV was initiated, thus, receiving a total dose of 200 mg/kg. The indications for MV after INSURE were acute respiratory insufficiency, poor oxygenation with an a/A-ratio <0.15, and respiratory acidosis (pH < 7.20) despite optimal nCPAP pressures. The indication for extubation after MV was a FiO_2_ of less than 0.40, peak inspiratory pressures less than 20–22 cm H_2_O and set mechanical ventilation frequency less than 25–30 minute^-1^. Acute neonatal anemia was usually transfused with 10–15 ml/kg packed red blood cells over 3 to 4 hours if hemoglobin was below 8.0 mmol/l.

Continuous variables were dichotomized prior to analyses using cut-off points regarded as clinically relevant. In the primary analyses, we estimated crude odds ratios (OR) for each predictor for the risk of INSURE failure using INSURE success as reference. The estimates were adjusted for co-variates chosen a priori: gestational age at birth (days), gender, and birth year in three groups (1998–2002, 2003–2006, 2007–2010). ORs are presented with 95% confidence intervals (CI). Gestational age was assessed in four groups (<26 weeks, 26 + 0 to 27 + 6 weeks, 28 + 0 to 29 + 6 weeks, and 30 + 0 to 31 + 6 weeks) using 30 + 0 to 31 + 6 weeks as the reference. We fitted all adjusted models using robust standard errors to take into account the clustering of siblings. In the secondary analyses, we compared the long MV group with the INSURE success group.

We compared the post-surfactant characteristics in the INSURE failure group, the short MV group, and the long MV group with the INSURE success group; Wilcoxon rank sum test was used for non-normally distributed continuous variables and logistic regression for dichotomous variables. In case of missing values, a complete case analysis was performed. The significance level was a 2-sided p value < 5%. The analyses were defined a priori. The statistical analyses were performed using STATA/SE 12.1 software (StataCorp LP, Texas, USA).

## Results

**Figure 1:**
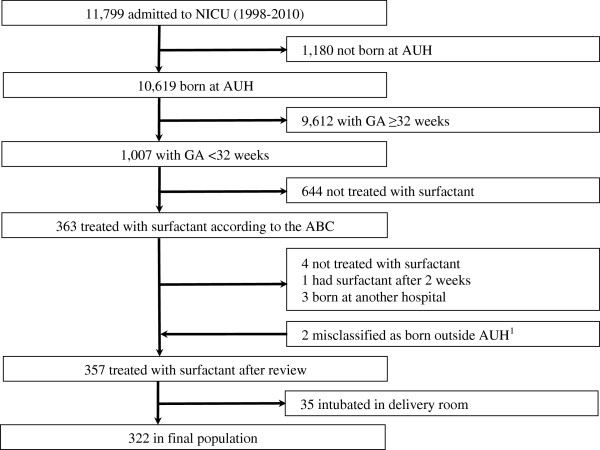
**Study population.** Abbreviations: AUH, Aarhus University Hospital; GA, gestational age; NICU, neonatal intensive care unit. ^1^74 neonates were born outside AUH according to the Aarhus Birth Cohort, but fulfilled the other inclusion criteria. Two of these neonates were included as they were actually born at AUH.

The study population consisted of 322 neonates born before 32 weeks gestation, treated with surfactant, and born 1998–2010 (Figure 1). Characteristics of the study population are presented in Table 1. In total, 31% (n = 100) had INSURE success, 10% (n = 33) had INSURE failure, 10% (n = 31) needed short MV, and 49% (n = 158) needed long MV.

**Table 1:** Characteristics of 322 surfactant treated neonates born before 32 weeks gestation, 1998–2010

**Characteristics**	**Study population (n = 322)**
Treatment group, n (%)^1^	
INSURE success	100 (31)
INSURE failure	33 (10)
Short MV	31 (10)
Long MV	158 (49)
Duration of intubation at surfactant, median (IQR), h^1^	
INSURE success	0.3 (0–0.5)
INSURE failure	0.5 (0–0.7)
Short MV	15.4 (8.0–19.7)
Long MV	57.3 (33.5–114.2)
Gestational age, n (%)^1^	
<26 + 0 weeks	70 (22)
26 + 0 – 27 + 6 weeks	92 (29)
28 + 0 – 29 + 6 weeks	102 (32)
30 + 0 – 31 + 6 weeks	58 (18)
Birth weight, n (%)^1^	
<750 g	53 (17)
750–999 g	104 (32)
1000–1249 g	80 (25)
≥1250 g	84 (26)
Small for gestational age, n (%)^1^	134 (42)
Gender, n (%), male^1^	190 (59)
Temperature at admission to NICU, mean (SD), °C^2^	36.1 (0.9)
Parity, n (%)^1^	
Nulliparous	190 (61)
Primiparous	85 (27)
Multiparous	37 (12)
Multiple pregnancies, n (%)^1^	
Singletons	187 (58)
Twins	119 (37)
Triplets	16 (5)
Antenatal steroids, n (%)^1^	285 (90)
Cesarean delivery, n (%)^1^	214 (66)
Preeclampsia, n (%)^2^	59 (20)
Prolonged rupture of membranes >18 hours, n (%)^2^	26 (9)
Maternal age at delivery, mean (SD), years^1^	29.9 (4.7)

*Abbreviations:* h, hours; IQR, interquartile range; long MV, mechanical ventilation for more than 24 hours; NICU, neonatal intensive care unit; short MV, mechanical ventilation for less than 24 hours.

Percentage of missing values for each characteristic: ^1^≤ 5%, and ^2^≤ 10%.

The predictors for INSURE failure (primary analyses) are shown in Table 2. For each 2-week decrease in gestational age, the risk of INSURE failure increased with an adjusted OR of 1.8 (95% CI: 1.2–2.8). Hemoglobin below 8.5 mmol/l was also associated with an increased risk of INSURE failure. The predictors for long MV (secondary analyses) are shown in Table 2. For each 2-week decrease in gestational age, the risk of long MV increased with an adjusted OR of 3.1 (95% CI: 2.3–4.3). After adjustment, Apgar at 5 minutes below 7, FiO_2_ above 0.50, pCO_2_ above 7 kPa (~53 mmHg), lactate above 2.5 mmol/l, pH below 7.3, inotropes given prior to intubation, and surfactant given within 5 hours after birth were all associated with the need for long MV. Preeclampsia reduced the risk of long MV.

**Table 2:** Predictors for an unsuccessful INSURE procedure in 291 neonates born before 32 weeks gestation, 1998–2010

		**Primary analyses** **INSURE failure vs. INSURE success**			**Secondary analyses** **Long MV vs. INSURE success**		
**Predictors**	**INSURE success, n (%) (n = 100)**	**INSURE failure, n (%) (n = 33)**	**Crude OR**	**Adjusted OR (95% CI)**^**1**^	**Long MV, n (%) (n = 158)**	**Crude OR**	**Adjusted OR (95% CI)**^**1**^
Gestational age^2^							
30 + 0 – 31 + 6 weeks (base)	34 (34)	3 (9)	1.0	1.0	11 (7)	1.0	1.0
28 + 0 – 29 + 6 weeks	40 (40)	16 (49)	4.5	4.8 (1.3–18.0)*	34 (22)	2.6	3.1 (1.3–7.5)*
26 + 0 – 27 + 6 weeks	18 (18)	8 (24)	5.0	4.9 (1.1–21.2)*	59 (37)	10.1	11.4 (4.6–28.6)*
– 25 + 6 weeks	8 (8)	6 (18)	8.5	9.9 (1.8–55.6)*	54 (34)	20.9	26.3 (9.5–72.5)*
Birth weight <1000 g^2^	27 (27)	10 (30)	1.2	0.3 (0.1–1.2)	105 (67)	5.5	1.6 (0.8–3.3)
Small for gestational age^2^	42 (42)	12 (36)	0.8	0.9 (0.4–2.0)	64 (41)	1.0	1.3 (0.7–2.3)
Gender (male)^2^	57 (57)	19 (58)	1.0	1.4 (0.6–3.2)	97 (61)	1.2	1.4 (0.7–2.6)
Umbilical cord pH <7.3^5^	26 (35)	6 (24)	0.6	0.7 (0.2–2.1)	42 (36)	1.0	1.1 (0.5–2.4)
Umbilical cord SBE < -3 mmol/l^6^	18 (26)	5 (24)	0.9	0.6 (0.2–2.0)	46 (42)	2.0	1.3 (0.6–2.8)
Apgar at 1 minute <7^2^	30 (30)	8 (25)	0.8	0.5 (0.2–1.3)	76 (48)	2.2	1.7 (0.9–3.3)
Apgar at 5 minutes <7^2^	4 (4)	2 (6)	1.6	1.4 (0.2–10.6)	23 (15)	4.1	4.7 (1.0–20.9)*
Temp at admission to NICU <35.5°C^3^	13 (14)	4 (14)	1.0	0.7 (0.2–2.7)	36 (25)	2.0	1.1 (0.5–2.7)
Surfactant given <5 hours after birth^2^	24 (24)	13 (39)	2.1	1.3 (0.5–3.5)	104 (66)	6.1	3.3 (1.7–6.2)*
a/A-ratio <0.20^4^	33 (34)	5 (17)	0.4	0.6 (0.2–1.8)	38 (31)	0.9	1.3 (0.6–2.7)
FiO_2_ > 0.50^3^	31 (33)	9 (29)	0.8	1.3 (0.5–3.6)	74 (50)	2.0	2.7 (1.3–5.3)*
pO_2_ < 7.5 kPa (~56 mmHg)^5^	17 (20)	5 (17)	0.8	0.8 (0.2–2.9)	28 (26)	1.4	1.1 (0.5–2.6)
pCO_2_ > 7 kPa (~53 mmHg)^4^	16 (18)	10 (33)	2.2	2.0 (0.7–5.8)	62 (50)	4.4	4.3 (1.9–9.6)*
pH <7.3^6^	30 (43)	13 (54)	1.5	1.8 (0.7–4.9)	65 (67)	2.6	3.9 (1.7–8.8)*
SBE < -3 mmol/l^6^	20 (29)	6 (25)	0.8	0.6 (0.2–1.9)	48 (49)	2.4	1.7 (0.7–3.8)
Hemoglobin <8.5 mmol/l^6^	2 (3)	5 (21)	8.6	5.1 (1.0–25.8)*	14 (15)	5.8	4.8 (0.7–34.7)
Lactate >2.5 mmol/l^6^	25 (37)	6 (25)	0.6	0.6 (0.2–1.9)	54 (59)	2.4	2.7 (1.3–5.7)*
Mean blood pressure < gestational age^6^	9 (13)	2 (12)	0.9	1.0 (0.2–6.9)	16 (17)	1.4	1.7 (0.6–5.2)
Inotropes prior to intubation^2^	2 (2)	1 (3)	1.5	0.9 (0.2–5.2)	17 (11)	5.9	8.8 (1.2–66.7)*
Nulliparity^2^	59 (60)	23 (72)	1.7	1.8 (0.7–4.8)	87 (58)	0.9	0.9 (0.5–1.6)
Multiple pregnancy^2^	45 (45)	16 (48)	1.2	0.9 (0.4–2.1)	66 (42)	0.9	0.6 (0.3–1.1)
Preeclampsia^3^	24 (26)	8 (25)	0.9	1.2 (0.4–3.2)	18 (12)	0.4	0.4 (0.2–0.8)*
Prolonged rupture of membranes^3^	8 (8)	1 (3)	0.4	0.2 (0.0–2.2)	16 (11)	1.4	1.1 (0.4–3.4)
Maternal age at delivery >30 years^2^	48 (48)	16 (48)	1.0	1.3 (0.5–3.0)	86 (55)	1.3	1.6 (0.9–3.1)
No antenatal steroids^2^	9 (9)	4 (13)	1.4	1.4 (0.4–5.4)	14 (9)	1.0	0.8 (0.3–2.0)
Cesarean delivery (vs. vaginal)^2^	72 (72)	23 (70)	0.9	1.5 (0.6–4.0)	95 (60)	0.6	1.0 (0.5–1.9)

*Abbreviations:* a/A-ratio, arterial to alveolar oxygen tension ratio; FiO_2_, fraction of inspired oxygen; long MV, mechanical ventilation for more than 24 hours; pCO_2_, partial pressure of carbon dioxide; pO_2_, partial pressure of oxygen; SBE, standard base excess; temp, temperature.

*P < 0.05.

^1^Adjusted for gestational age, gender, and birth year using robust standard error correction for clustering. Gestational age was adjusted for gender and birth year. Gender was adjusted for gestational age and birth year.

Percentage of missing values for each predictor: ^2^≤ 5%, ^3^≤ 10%, ^4^≤ 20%, ^5^≤ 30%, and ^6^≤ 40%.

The post-surfactant characteristics for the four treatment groups are shown in Table 3. The INSURE success group had characteristics similar to the short MV group, and the INSURE failure group had characteristics similar to the long MV group with respect to the following respiratory variables: pneumothorax, duration of MV, duration of nCPAP, and duration of oxygen supplementation.

**Table 3:** Post-surfactant characteristics for 322 neonates born prior to 32 weeks gestation, 1998–2010

**Post-surfactant characteristics**	**Treatment group**			
	**INSURE success (n = 100)**	**INSURE failure (n = 33)**	**Short MV (n = 31)**	**Long MV (n = 158)**
CRIB-score, median (IQR)^3^	2 (1–2)	2 (2–6)*	2 (2–5)*	6 (2–8)*
CRIB-score > 4, n (%)^3^	6 (8)	9 (32)*	9 (36)*	79 (66)*
Given < 6 hours after surfactant:				
Inotropes, n (%)^2^	2 (2)	5 (15)*	1 (3)	56 (35)*
Packed red blood cells, n (%)^2^	6 (6)	3 (9)	1 (3)	18 (11)
Volume expansion, n (%)^2^	10 (10)	5 (15)	5 (16)	72 (46)*
Antibiotics ≥ 7 days, n (%)^2^	17 (17)	6 (18)	12 (39)*	61 (39)*
Pneumothorax^2^	2 (2)	8 (25)*	1 (3)	35 (23)*
Necrotizing enterocolitis, n (%)^2^	10 (10)	8 (24)*	4 (13)	56 (36)*
Clinical suspicion or x-ray changes	9	5	4	50
Surgery	1	3	0	6
Patent ductus arteriosus on first echocardiography, n (%)^2^	33 (33)	16 (48)	13 (42)	71 (45)
No treatment	19	11	11	49
NSAID/surgery	14	5	2	22
Intraventricular hemorrhage, n (%)^2^	38 (38)	12 (36)	7 (23)	73 (46)
Grades I–II	34	7	3	32
Grades III–IV	4	5	4	41
Periventricular leukomalasia, n (%)^2^	4 (4)	1 (3)	2 (6)	15 (9)
BPD, n (%)^1,4^	12 (18)	5 (26)	4 (24)	27 (46)*
Retinopathy of prematurity grade III, n (%)^2^	3 (3)	1 (3)	0 (0)	12 (8)
MV days, median (IQR)^2^	0.01 (0.00–0.03)	3.26 (1.59–4.45)*	0.70 (0.33–0.92)*	3.26 (1.77–6.98)*
CPAP days, median (IQR)^2^	17.5 (9.3–31.5)	21.5 (10.3–33.1)	19.0 (6.7–34.1)	22.4 (1.2–36.2)
Oxygen days, median (IQR)^2^	6 (2–20)	10 (4–29)	5 (0–21)	15 (3–40)*
Neonatal death < 28 days, n (%)^2^	1 (1)	4 (12)*	2 (6)	41 (26)*

*Abbreviations:* BPD, bronchopulmonary dysplasia; CRIB, clinical risk index for babies; IQR, interquartile range; long MV, mechanical ventilation for more than 24 hours; short MV, mechanical ventilation for less than 24 hours.

*P < 0.05 for the association with INSURE success.

^1^60 neonates died before 36 completed weeks and were thus not included in the analysis.

Percentage of missing values for each post-surfactant characteristic: ^2^ ≤ 5%, ^3^23%, and ^4^40%.

## Discussion

The risk of INSURE failure and long MV increased with decreasing gestational age at birth. In addition, the primary analyses showed that low hemoglobin prior to surfactant was a predictor for INSURE failure. Furthermore, the secondary analyses showed that low Apgar at 5 minutes, high FiO_2_, high pCO_2_, high lactate, low pH, inotropes given prior to intubation, and surfactant administered within 5 hours after birth were associated with an increased risk of long duration of MV, whereas preeclampsia reduced the risk.

Cherif et al. 2008 and Dani et al. 2010 previously assessed predictors of INSURE failure defined as re-intubation within 72 hours among neonates of 27–34 weeks’ gestation and below 30 weeks’ gestation, respectively [5,6]. A short gestational age was not associated with INSURE failure in these studies, but birth weight was associated with INSURE failure even after adjustment for potential confounding variables [5,6]. Due to lack of difference in gestational age between the two groups, gestational age was not included in the multivariate model [5,6]. We found no association between birth weight or being SGA and INSURE failure after adjustment for gestational age.

The two previous studies that have identified neonates with INSURE failure found that they had more severe RDS in terms of low a/A-ratio, high pCO_2_, and low pO_2_/FiO_2_[5,6]. In the present study, similar predictors were found for long duration of MV, but not for INSURE failure. This indicates that the neonatologist involved in our study opted to treat neonates with more severe RDS with MV. Thus, the influence of these specific predictors by may be underestimated if INSURE failure is studied as the outcome rather than considering unsuccessful INSURE as a combination of INSURE failure and long MV.

A low hemoglobin concentration was associated with INSURE failure. An explanation could be that the insufficient oxygen delivery to peripheral tissues increased lactate and decrease pH. However, high lactate and low pH were not associated with INSURE failure. Another speculation might be that INSURE failure is associated with poor perfusion and/or hypotension, and if treated with volume expansion it may cause a seemingly low hemoglobin concentration due to dilution. However, low mean arterial blood pressure was not associated with INSURE failure. Thus, no obvious explanation was found for this observation. Only two neonates in the INSURE success group had low hemoglobin, and 32% had missing values for hemoglobin: thus, the association could be due to chance. However, in univariate analysis, Cherif et al. also found that a low hemoglobin concentration increased the risk of INSURE failure [5]. This potentially strong association deserves further investigation.

Preeclampsia seemed to reduce the risk for a long duration of MV. This was rather unexpected because preeclampsia has previously been associated with an increased risk of BPD in neonates at 23 to 32 weeks gestation [19]. We consider that neonates born of mothers without preeclampsia may likely be born preterm for some other more severe cause, e.g., infection. This may in turn explain the potentially spurious protective association between preeclampsia (relatively benign for the neonate) and long MV. The technical term for this phenomenon is collider bias, which may arise from conditioning on gestational age [20].

The attending neonatologist may choose not to use INSURE and keep the most premature or ill neonates on MV; neonates that would most likely fail the respiratory challenge of INSURE. Neonates on MV are routinely evaluated for MV weaning at least every 24 hours. We assumed that all mechanically ventilated neonates that would have been INSURE successes if INSURE had been attempted were extubated within 24 hours. Thus, the remaining neonates (MV for more than 24 hours) would all have been INSURE failures, had INSURE been attempted. The predictors for long MV might be interpreted as predictors of INSURE failure. In accordance with this, the predictors for long MV in the present study were comparable with the predictors for INSURE failure found by the two previous studies [5,6]. Furthermore, the post-surfactant characteristics of the population (Table 3) were similar in the INSURE success group and the short MV group, whereas in INSURE failure group and the long MV group, they were similar. This supports the above assumption, and we thus might consider predictors for long MV as predictors for INSURE failure. Consequently, almost all neonates born at our tertiary center and treated with surfactant were included in the analyses. This reduces selection bias, increases power, and increases the external validity.

The neonatologist’s decision on whether or not to treat the patient with INSURE is the greatest limitation in the current study. During the first 24 hours after surfactant administration, neonates on MV were evaluated for MV weaning. There might be a risk that the neonate was kept on MV after this evaluation, not because the neonate needed MV, but simply because INSURE failure was suspected at the initial surfactant administration. Consequently, the neonate could be kept on MV for more than 24 hours, even though the neonate would have been an INSURE success if INSURE had been performed. This could lead to spurious associations with long MV. However, the predictors for long MV were similar to predictors for INSURE failure in the former studies [5,6]. The predictors for long MV might overestimate the true effect because these neonates are usually in poorer condition than those in the INSURE failure group, which is indicated by the more severe predictors (Table 2) and post-surfactant characteristics (Table 3). The attending neonatologist’s decision to treat a neonate according to INSURE or MV after surfactant tends to cause an underestimation of the effect of the predictors for INSURE failure if the neonatologist suspects the same set of predictors as those we have evaluated. We defined INSURE as intubation for less than 2 hours without the need for MV. This cut-off point was used to take into account the variability in time noted in the medical records by the neonatologists. This may introduce non-differential information bias, causing bias toward the null. Finally, a high proportion of values were missing with regard to some predictors (Table 2).

In Cherif et al., the pre-intubation medication was morphine [5]. Information on medication was unavailable in the study by Dani et al. [6]. Morphine is a long-acting drug that may increase the risk of INSURE failure and the need for MV, even though the effect is antagonized with naloxone [21]. The medications used in our population included both thiomebumal and morphine, which may account for some of the need for continued MV after surfactant. However, this is only speculative because thiomebumal has never been evaluated with respect to the effect on INSURE failure or need for continued MV after surfactant [21]. We emphasize that the same pre-intubation medications were given to all neonates, and therefore, this could not have confounded the results. Thus, the pre-intubation medication does not violate the internal validity of the assessed predictors.

The observational design of this study implies that causal inference cannot be made. However, if the distribution of confounders is the same in other settings, the identified predictors will also predict unsuccessful INSURE in future populations, even though the associations are not causal. Whether the identified high-risk neonates would have benefit of being treated with MV immediately after surfactant instead of INSURE has to be tested in a future randomized controlled trial. The identified predictors for an unsuccessful INSURE procedure may be used to guide a risk stratification for such a future randomized controlled trial.

## Conclusions

We observed an increased risk of INSURE failure in very preterm neonates with decreasing gestational age at birth and a low hemoglobin concentration prior to surfactant administration. Predictors for long duration of MV might also predict INSURE failure. These predictors were low gestational age, low Apgar at 5 minutes, high FiO_2_, high pCO_2_, high lactate, low pH, inotropes given prior to intubation, and surfactant administered within 5 hours after birth. Preeclampsia reduced the risk of long MV. Our results can be used to identify neonates at high risk of an unsuccessful INSURE procedure, which may guide risk stratification in the clinical setting and planning of a future randomized controlled trial to assess whether these high-risk neonates would benefit from treatment with MV immediately after surfactant instead of INSURE.

## Abbreviations

RDS: Respiratory distress syndrome; INSURE: INtubation-SURfactant-Extubation; BPD: Bronchopulmonary dysplasia; MV: Mechanical ventilation; nCPAP: Nasal continuous positive airway pressure; a/A-ratio: Arterial to alveolar oxygen tension ratio; pO_2_: Partial pressure of oxygen; pCO_2_: Partial pressure of carbon dioxide; FiO_2_: Fraction of inspired oxygen; NICU: Neonatal intensive care unit; AUH: Aarhus University Hospital; ABC: Aarhus Birth Cohort; GA: Gestational age; SGA: Small for gestational age; SBE: Standard base excess; CRIB-score: Clinical risk index for babies; OR: Odds ratio; CI: Confidence interval.

## Competing interests

The authors declare that they have no competing interests.

## Authors’ contributions

TBH conceptualized and designed the study. NB drafted the initial protocol, made the data collection instrument, collected data, performed data management and analyses, drafted and revised the initial manuscript. LVP collected data for the Aarhus Birth Cohort. AS, MSJ, and TBH supervised and critically revised the protocol, and supervised the design of the data collection instrument. AS, MSJ, LVP, and TBH supervised the data collection and analyses, and critically reviewed and revised the manuscript. All authors approved the final manuscript as submitted, and have agreed to be accountable for all aspects of the work.

## Pre-publication history

The pre-publication history for this paper can be accessed here:

http://www.biomedcentral.com/1471-2431/14/155/prepub
